# Clinical supervision and feedback in Indianoceany medical education: the challenge of socio-cultural interactions

**DOI:** 10.5116/ijme.67ce.e2e6

**Published:** 2025-04-16

**Authors:** Rémi Girerd, Arnaud Winer, Andriambelo Tovohery Rajaonera, Morgan Jaffrelot, Marie Claude Audetat

**Affiliations:** 1 Indian Ocean Health Simulation Center, CHU de la Réunion, Saint Pierre, France; 2 Faculty of Medicine, University of Reunion Island, Saint Pierre, La Réunion, France; 3 Faculty of Medicine, Antananarivo, University of Madagascar, Madagascar; 4 Department of Anesthesiology and Intensive Care, Université Laval, Québec, QC, Canada; 5 Faculty of Medicine, University of Geneva, UDREM and Primary Care Unit (IuMFE), Switzerland

**Keywords:** Clinical supervision, experiential learning, clinical teaching environment, Indianoceany, healthcare sciences

## Introduction

In the healthcare sciences, particularly in medical education, the focus extends beyond the mere acquisition of knowledge. It encompasses the progressive development of all the skills learners will require in their professional lives, especially in clinical contexts. In this regard, clinical supervision, and particularly the provision of feedback, are well-established methods that enable learners to continuously enhance their performance.^1^ Although the clinical teaching environment offers little control over case flow, the variety of problems, or time management, it holds immense pedagogical potential for experiential learning and the modelling of best practices. Thus, healthcare professionals must learn to balance two critical roles: that of clinician, responsible for patients, and that of supervisor or educator, tasked with guiding learners in the development of their professional skills.

### Medical education experience in Madagascar - Indianoceany

Madagascar is a country located in the southwern Indian Icean, also called the Indianoceany. The Indian Commission Ocean defines the Indianoceany as the island nations and territories situated in the southwestern Indian Ocean, including Comoros, Madagascar, Mauritius, Réunion (France), and Seychelles.^2^ Each island has a distinct and varied history and culture. Although nearly 90% of the population does not speak French as a first language, French remains the cultural, geopolitical, and economic choice. Réunion Island, shaped by "a history of slavery, servitude, colonialism, cross-cultural encounters, assimilation, resistance, and hybridisation," has long dealt with the challenges of managing cultural diversity within its population, which has roots in Europe, Asia, and Africa.^3^ Consequently, each country trains its healthcare professionals locally, within its own cultural context, often with the support of regional or international partnerships. For instance, Mauritius collaborates with India and Canada, while Madagascar enjoys a privileged partnership with France, including Réunion Island. Healthcare professionals from Reunion Island regulary contribute to fostering both clinical and pedagogical skills (for example to do a relevant feedback), in development in practising healthcare professionals through continous education, as well as in learners from various medical and paramedical fields.

In our research on clinical supervision practices in Madagascar, as well as the beliefs and perceptions associated with it, our preliminary findings point to a not-evidence-based-medical-education-science approach to pedagogy, which aligns with Sari's conclusions.^4^ Several significant themes emerge from this intuitive perspective, including a considerable gap between teachers' expressed concern for their students' well-being and students' perceptions of distance and severity. Dominant beliefs are closely tied to the clinician's role and reflect a transmissive, hierarchical model of supervision ("If you are a good clinician, you are a good teacher"; "We are somewhat like their parents"). Participants in our study (mainly medical and nursing educators) express a strong desire to further develop their pedagogical skills.

It seems crucial to support this motivation in order to value and reinforce the role of these healthcare professionals as supervisors.

### Challenges and perspectives

Recent research in health sciences pedagogy underscores the importance of considering the sociocultural nature of interactions during supervision and feedback.^4^ Studies have demonstrated that feedback does not always fulfil its intended purpose of stimulating learning, but can sometimes provoke counterproductive emotional responses, such as anxiety, frustration, or demotivation. Recent literature on supervision and feedback therefore highlights the critical role and responsibility of educators in forming a pedagogical alliance with their learners, establishing a safe learning environment, and promoting reflection on their performance.^5^

The concept of culture has been defined as an "integrated pattern of learned beliefs and behaviours shared among groups, which includes thoughts, communication styles, modes of interaction, views on roles and relationships, values, practices, and customs".^6^ While these cultural aspects have been extensively explored in the context of the caregiver-patient relationship, much less attention has been paid to the supervisor-learner dynamic.

However, recent studies by Sari et al. have revealed that in countries with a strong hierarchical tradition, the supervisor-learner relationship, particularly within the feedback framework, may take on a "parent-child" connotation. Feedback in these contexts often focuses on learners' mistakes and limitations and tends to be prescriptive, rather than fostering dialogue or encouraging reflection on practices and the cognitive processes involved.^4^ Their findings also indicate a tendency towards teacher-centred, rather than learner-centred, instruction.

To date, the few studies on the significance of cultural dimensions in clinical supervision and feedback have primarily explored differences between Anglo-Saxon and Asian or Indonesian cultures. Reflections on the development of cultural competencies in educators remain in their infancy. Several cultural competence assessment tools exist and include different aspects such as knowledge of other cultures, communication skills, cultural sensibility and respect, and need to be adapted in every context.^7^

It is therefore vital to deepen our understanding of the perspectives of both trainers and learners within all the contexts, in order to better train healthcare providers, improve supervision practices, and ultimately enhance healthcare outcomes.

In Indianoceany, and more broadly across the African continent, the socio-cultural dimension — along with the requisite development of cultural competence in each country — must be addressed through further research, benefiting both the countries involved and the clinician-educators engaged in these regions.

### Conflict of Interest

The authors declare that they have no conflicts of interest.
